# Significant reduction of carbon stocks and changes of ecosystem service valuation of Indian Sundarban

**DOI:** 10.1038/s41598-022-11716-5

**Published:** 2022-05-12

**Authors:** Biswajit Bera, Sumana Bhattacharjee, Nairita Sengupta, Pravat Kumar Shit, Partha Pratim Adhikary, Debashish Sengupta, Soumik Saha

**Affiliations:** 1 Department of Geography, Sidho-Kanho-Birsha University, Ranchi Road, P.O. Purulia Sainik School, Purulia, 723104 India; 2 Department of Geography, Jogesh Chandra Chaudhuri College (University of Calcutta), 30, Prince Anwar Shah Road, Kolkata, 700 033 India; 3 Department of Geography, Diamond Harbour Women’s University, Sarisha, 743368 India; 4 PG Department of Geography, Raja Narendralal Khan Women’s College (Autonomous), Vidyasagar University, Midnapore, 721102 India; 5 ICAR Indian Institute of Water Management, Bhubaneswar, Odisha 751023 India; 6 Department of Geology & Geophysics, Indian Institute of Technology (IIT), Kharagpur, West Bengal 721302 India

**Keywords:** Environmental sciences, Risk factors

## Abstract

The Sundarban mangrove or tidal influenced natural ecosystem is extremely productive and providing multiple goods and services to millions of people. In the last few decades, the quality and quantity of mangrove ecosystem are being deteriorated. The main objectives of this current research are (i) to investigate the ecosystem service values (ESVs) using a time series satellite data (1975, 2000 and 2020) and different unit values (ii) to analyze the dynamic pattern of carbon sequestration through InVEST model and (iii) determination of ESVs change hotspots by Getis-Ord Gi^*^ method. Here, mangrove forest has the highest ecosystem service value and highest carbon sinker. The total loss of ESVs was estimated 3310.79 million USD during last 45 years in Sundarban Biosphere Reserve (SBR) due to high natural and anthropogenic adversities. InVEST model also revealed that the total static carbon storage over the study area was 48.87, 46.65 and 43.33 Tg for the year 1975, 2000 and 2020 respectively. Total 6313944 mg/6.31Tg loss of carbon has been observed in the case of mangrove forest during the overall study period (1975–2020). So, illegal human encroachment should be strictly (through law and regulations) restricted within Sundarban mangrove ecosystem for the benefits of people.

## Introduction

Ecosystem services (ES) refer the convenience, benefits and bunch of services and goods from natural ecosystem or natural capital and these are indispensable for human well-being, livelihood and survival^1,2^. Ecosystem service valuation (ESV) signifies the monetary values on many ecosystem services that provided by the ecosystem as well as natural environment. It evaluates the market prices or values of common ecological units as well as different land use land cover classes that can eventually indicate us to realize the socio-environmental importance of these nature induced services and functions. It also helps to generate a green economy in a region^3^. In 1997, the global ESVs (ecosystem service values) were estimated around 33 trillion USD (United State Dollar) per year that was significantly greater than the global GDP (gross domestic product) on the same period^4^. Costanza et al.^4^ again estimated the global ecosystem service values in 2011 (125 trillion USD) using the previous method^3^ along with updated value. Quantification of the global changing pattern of ESVs is a momentous tool that can significantly build up a management approach regarding these natural capitals^5^.

The costal mangrove region is a completely different ecosystem region that can extensively provide various regulatory services (waste treatment, protection from storm surges and tropical cyclones, habitat provision etc.), provisional services (fish, timber and non-timber product, honey etc.), supporting services (biomass production) and cultural services^6^. Presently, several primary and secondary driving factors such as biophysical (climate change, changes in soil properties, plant inherent structure, composition etc.) and anthropogenic (land degradation, land use change etc.) are more responsible for the devaluation of ecosystem services^7,8^. Among the driving factors, human induced land use change is the most eventual factor for the reduction of ESVs^9^. Some previous studies have already analysed the superior impact of LULC change over the depreciation of ecosystem services in a regional or global scale^4,10^. The paradigm of ecosystem services had gained broader outlook in 2005 when the UN (United Nations) was published the MEA (Millennium Ecosystem Assessment) report. Within the period 2007–2010, a second preliminary action was taken by the UNEP (United Nation Environment Programme) that is known as The Economics of Ecosystems and Biodiversity (TEEB)^11^. The TEEB report was asserted by the media and this concept was distributed into a broader audience. Additionally, the World Business Council for Sustainable Development was actively taken an important part for the development of the ecosystem services concept^4^.

Presently, near about 60% ecosystem services are under threat due to unscientific exploration and exploitation of natural components by the humans^12^. Mangrove forests are mainly found along the tropical and sub-tropical coastlines. Total 75% tropical and sub-tropical countries in the world have been attributed with mangrove ecosystem^13^. Sundarban is the largest single tract mangrove forest region^14^. Most of the coastal communities are directly connected with different ESs. However, it is very challenging to measure the economic values of these bio-physical units mainly due to over or under estimation and the double counting^15^. Indian Sundarban consists of different land use land cover types. For proper valuation of ecosystem services, it has been classified into different ecosystem units and consequently government can apply necessary management steps for individual ecosystem units (Those are mangrove forest, settlement, sparse forest, waterbody, cropland and fallow land) in SBR. Presently, these natural reserve capitals are being gradually degraded due to present demand of various ESs and the overexploitation of these natural capitals particularly in coastal tracts^16^. Carbon sequestration indicates the diminishing process of carbon from atmosphere and stored into different carbon reservoirs in the ground.

Recent researchers have evaluated that the carbon sink effect is higher in mangrove ecosystem and mangrove forests are the carbon rich forest with an average of 1023 Mg carbon per hectares^17^. In the carbon-rich Sundarban ecosystem, the organic rich soil is accounted 49–98% carbon storage within 0.5–3 m depth^17^. The changing patterns of climate in Sundarban region have been observed within the last few decades. The temperature is higher in Sundarban region compared with the global changing trend. The surface temperature is rising at the rate of 0.5 °C/decade^18^. Health of the total environment of this coastal region has been drastically changed due to rapid conversion and modification of land use and land cover conditions within the biosphere region^19^.

The coastal mangrove acts as a great carbon sinker or reservoir with a rate of 2–4 times higher than a mature tropical or sub-tropical forest region^20^. The previous literatures have already shown that species diversity or variation has direct and indirect function composition on carbon storage in a terrestrial eco-unit. A recent study has revealed that mangrove forest of Sundarban can sequestrate about 4.71–6.54 Mg C ha^-1^ year^-1^ carbon^21^. The Sundarban region comprises with two eco-regions, swamps and mangroves but in the recent years these fresh water swamps became extinct due to speedy expansion of agricultural lands and fisheries^22^. Tropical forest regions have a significant role in maintaining the global carbon cycle and balance and it consists around 40% terrestrial NPP (net primary production)^23^. Mangrove forest communities are particularly found in the coastal and estuary region of tropical and sub-tropical climate and these forests play a significant role in socioeconomic and ecological pattern over the region by contributing coastline development and protection and act as nutrient filter and providing different material services^1,24^.

With the advancement of geospatial science and technology in the field of ESs studies, spatially explicit valuation, transfer and modelling are found to be useful to its cost and time benefits. Numerous approaches have been developed for the clear estimation of ecosystem services that significantly include the market price and benefit transfer approach^1,4,25^. In coastal areas, carbon mainly sinks into mangroves and tidal marshes (commonly refers as blue carbon) and considerably attracted the environmentalist^26^. Some relevant hypotheses have been considered regarding drop of carbon storage and changes of ecosystem service values for mangrove ecosystem of Indian Sundarban. Natural (coastal storm, sea level rise, increase of sea surface temperature and salinity etc.) as well as anthropogenic (human encroachment and reduction of sediment and sweet water supply) factors are maybe responsible for such rapid reduction of carbon storage and ecosystem service values. For such scientific study, the principal objectives are (i) to classify the entire SBR region into different ecosystem units (major components within the SBR ecosystem) (ii) to quantify spatially explicit ecosystem service values (ESVs) with reasons for temporal changes (iii) an approach has been taken to measure the temporal changes of static carbon storage and demarcation of ESVs hot spot and cold spot region within SBR region.

## Materials and methods

### Study area

**Figure 1:**
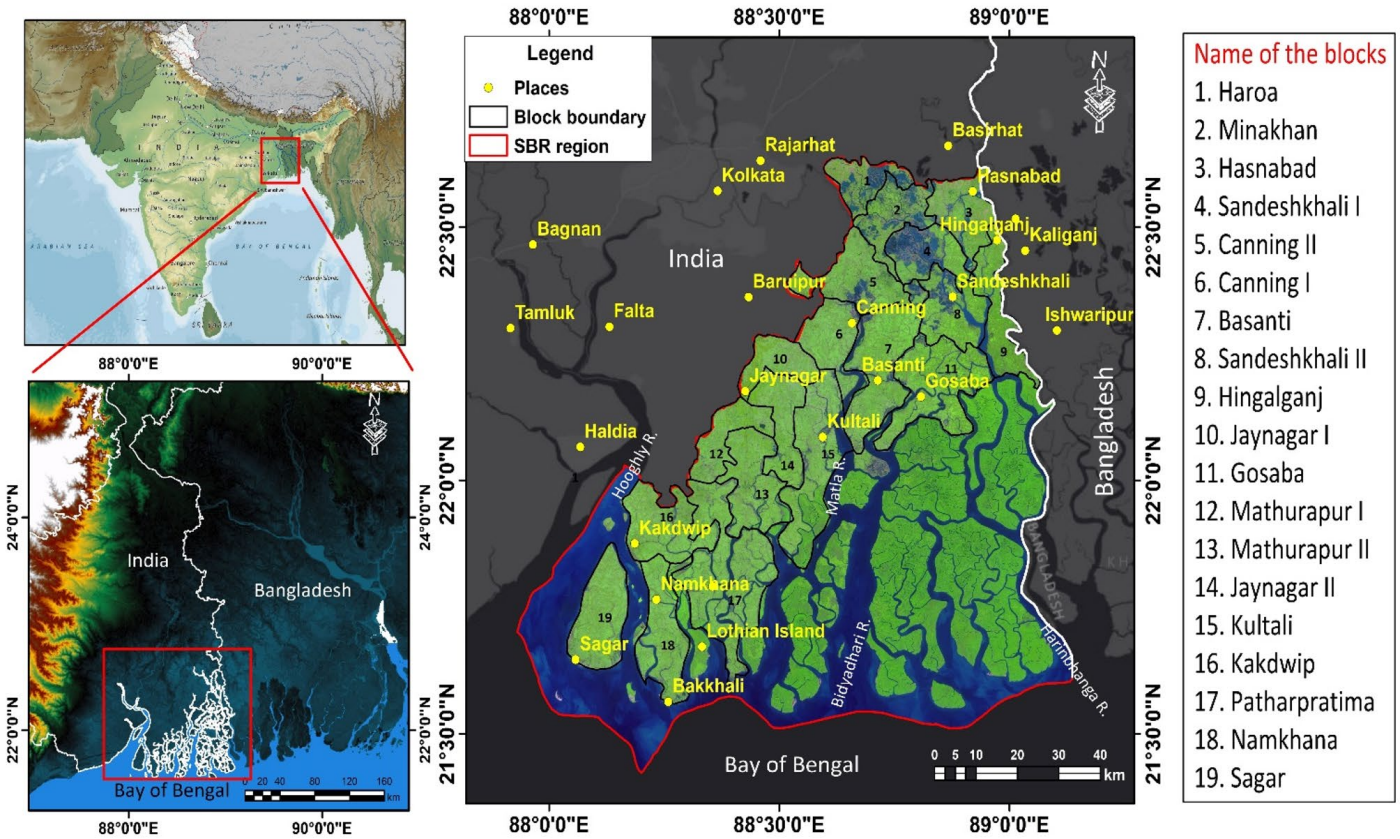
Geographical location of the Sundarban biosphere reserve. The maps were prepared using ArcGIS 10.3 software [https://desktop.arcgis.com].

The mangrove region of Sundarban is the world’s most extensive halophytic mangrove eco-region or area that covers around 3% entire land of world mangroves^27^. The Sundarban mangrove region is the significant coastal ecosystem (wetland and estuary ecosystem) that is restricted under the confluence zone of river Ganga, Meghna and Brahmaputra with a spatial extension of approximately 10,000 km^2^ area (Bangladesh and India share total 62% and 38% areas respectively) (Fig. 1). The inward or the landward limit of Indian Sundarban (21°32'N to 22°40'N and 88°05′E to 89°51′E) is bounded by the Dampier-Hodges line (Fig. 1). The Sundarban biosphere reserve (SBR) has different eco-sensitive areas (Sundarban tiger reserve, wildlife sanctuaries, Lothian Island, Holiday Island, Sundarban national park etc.)^15^. A unique physiography has been observed in this deltaic mangrove region that includes tidal inlets and creeks, sand beaches, sand flats, estuary, dune, mud flats, mangrove littoral swamp forest etc. The physiography of Sundarban region is most dynamic because this region is located in the near shore and foreshore region of Bay of Bengal and this region is under the transitional zone of two contrasting land masses (land and water) (Fig. 1). The Indian part of Sundarban area consists of total 102 islands (52 islands are habited and 48 are uninhabited whereas remaining two are eroded by the rising trend of ocean water)^28^. The Sundarban has been enlisted as world heritage site by UNESCO in 1997 due to its extraordinary ecological richness and biodiversity importance^18^.

### Assessment of land use land cover transformation within the time frame

**Figure 2:**
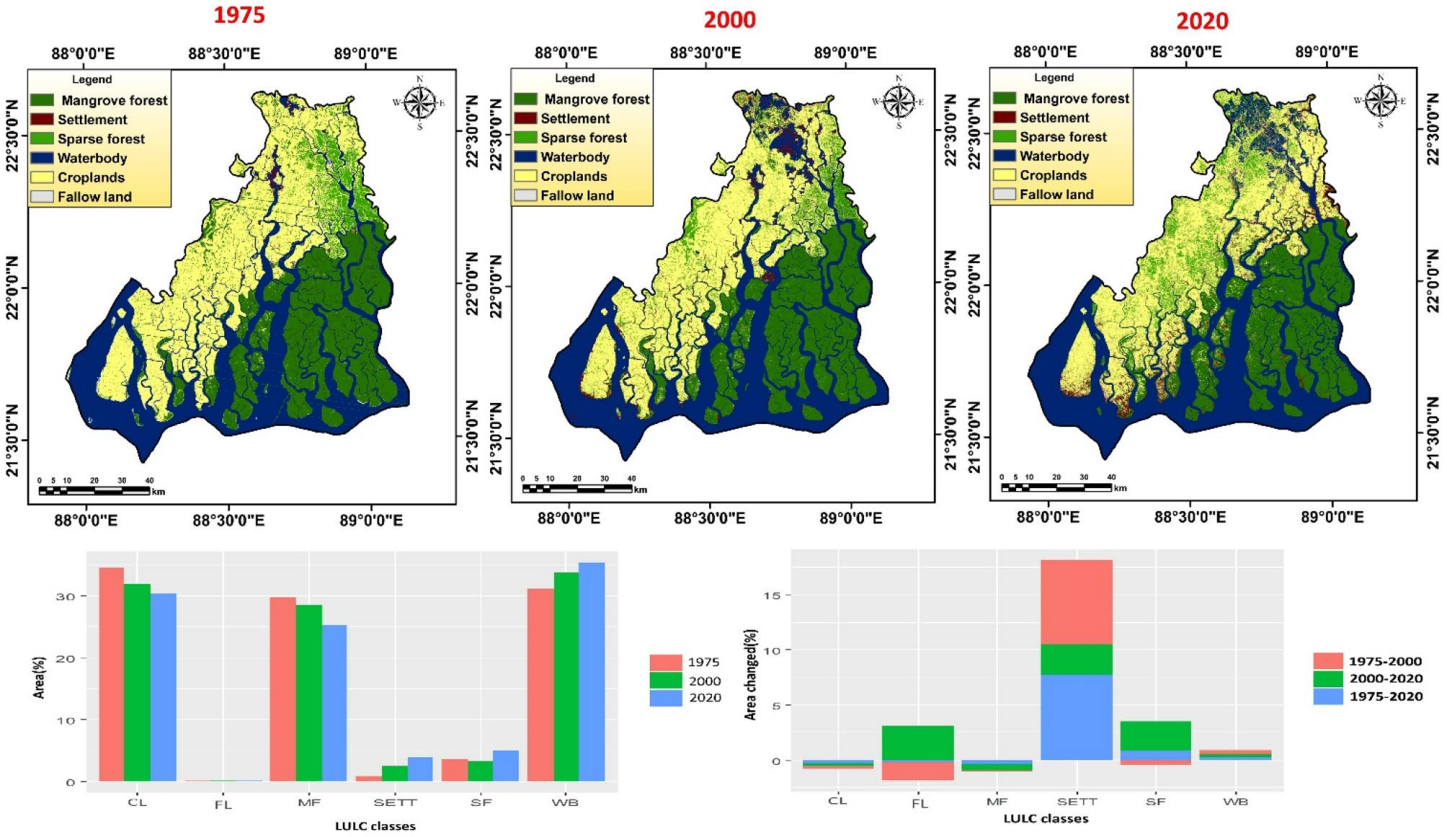
Land use land cover map (LULC) of various reference years (1975, 2000 and 2020) with respective LULC changes (*CL*  cropland, *FL*  fallow land, *MF*  mangrove forest, *SETT*  settlement, *SF*  sparse forest and *WB*  waterbody). The maps were prepared using ArcGIS 10.3 software [https://desktop.arcgis.com].

Total six Landsat images (two MSS (Multispectral scanner), two ETM + (Thematic mapper) and two OLI (Operational land imager)) have been used to classify the study region into different ecosystem unit for the year 1975, 2000 and 2020. MSS of 1975 (path/rows 148/44 and 148/45, 60 m spatial resolution and four spectral bands), ETM + of 2000 (path/rows 138/44 and 138/45, 30 m spatial resolution and eight spectral bands) and OLI_TIRS of 2020 (path/rows 138/44 and 138/45, 30 m spatial resolution and eleven spectral bands) have been retrieved from USGS website (Table S2). Two MSS images, two ETM + images and two OLI_TIRS have been combined separately in Erdas Imagine software. All satellite images are taken in pre monsoon period (Feb-May) due to cloudless observation. Dark object subtraction method has been used here and it is very much effective for haze reduction process. Maximum likelihood classification algorithm has been applied for classification of the entire study region into six different ecosystem units (water body including river/creeks, settlement, cropland, mangrove forest, sparse/open vegetation and fallow land) (Fig. 2). Total 250 random points have been created within the Sundarban biosphere reserve (SBR) and validate it through high accuracy Google Earth satellite image and an error matrix has been developed for accuracy assessment. The transformation intensity of each LULC classes has been measured as follows,

1 $$ Transformation \;intensity = \frac{{LULC_{end \;year} - LULC_{start \;year} }}{{LULC_{start \;year} }}*\frac{100}{{t \;period}} $$

where, ${LULC}_{end year} and{ LULC}_{start year}$ signify end and start time respectively and t means the time period.

### Estimation of ecosystem service values (ESVs)

**Figure 3:**
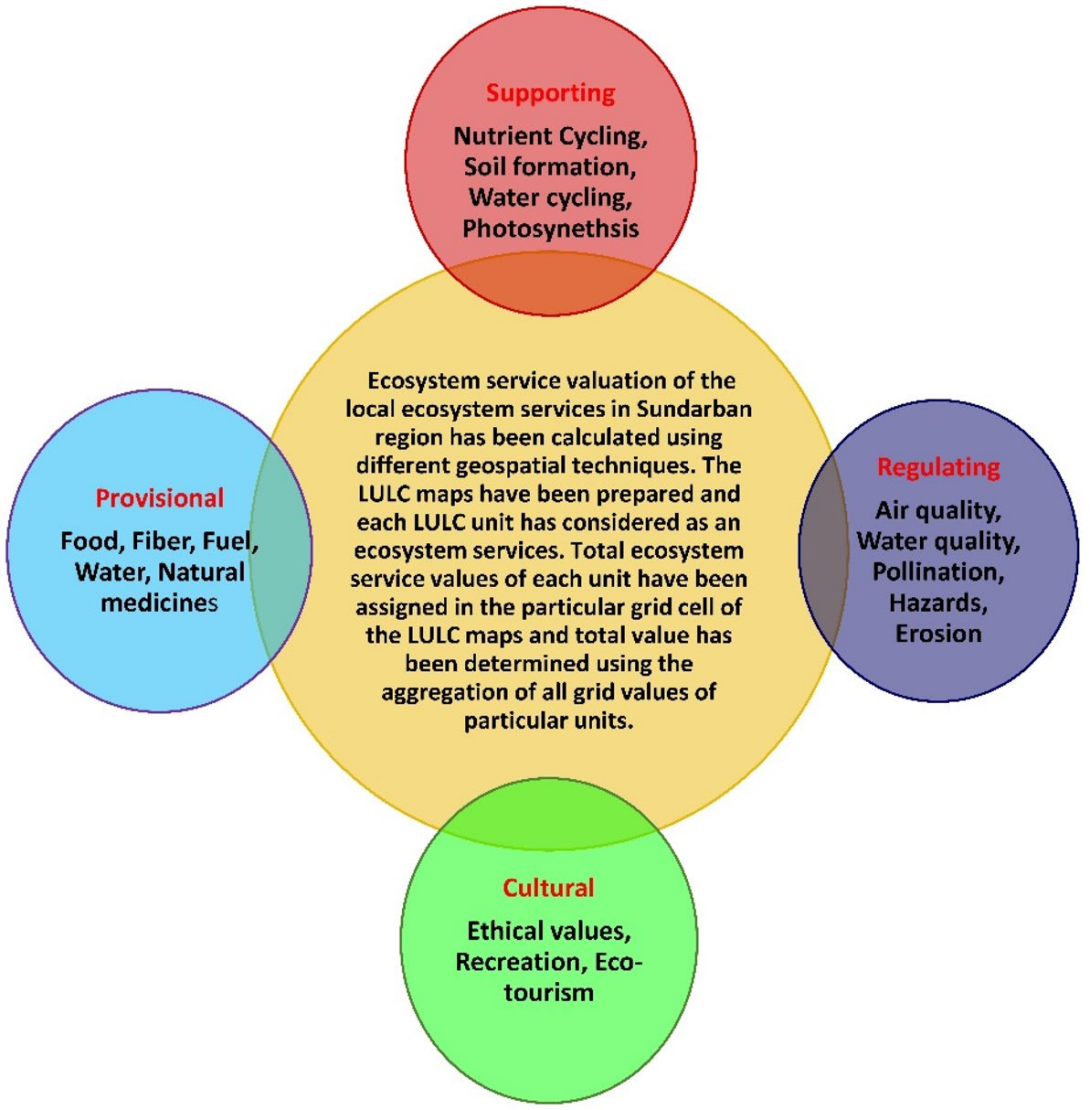
Graphical structure of the various ecosystem services.

**Figure 4:**
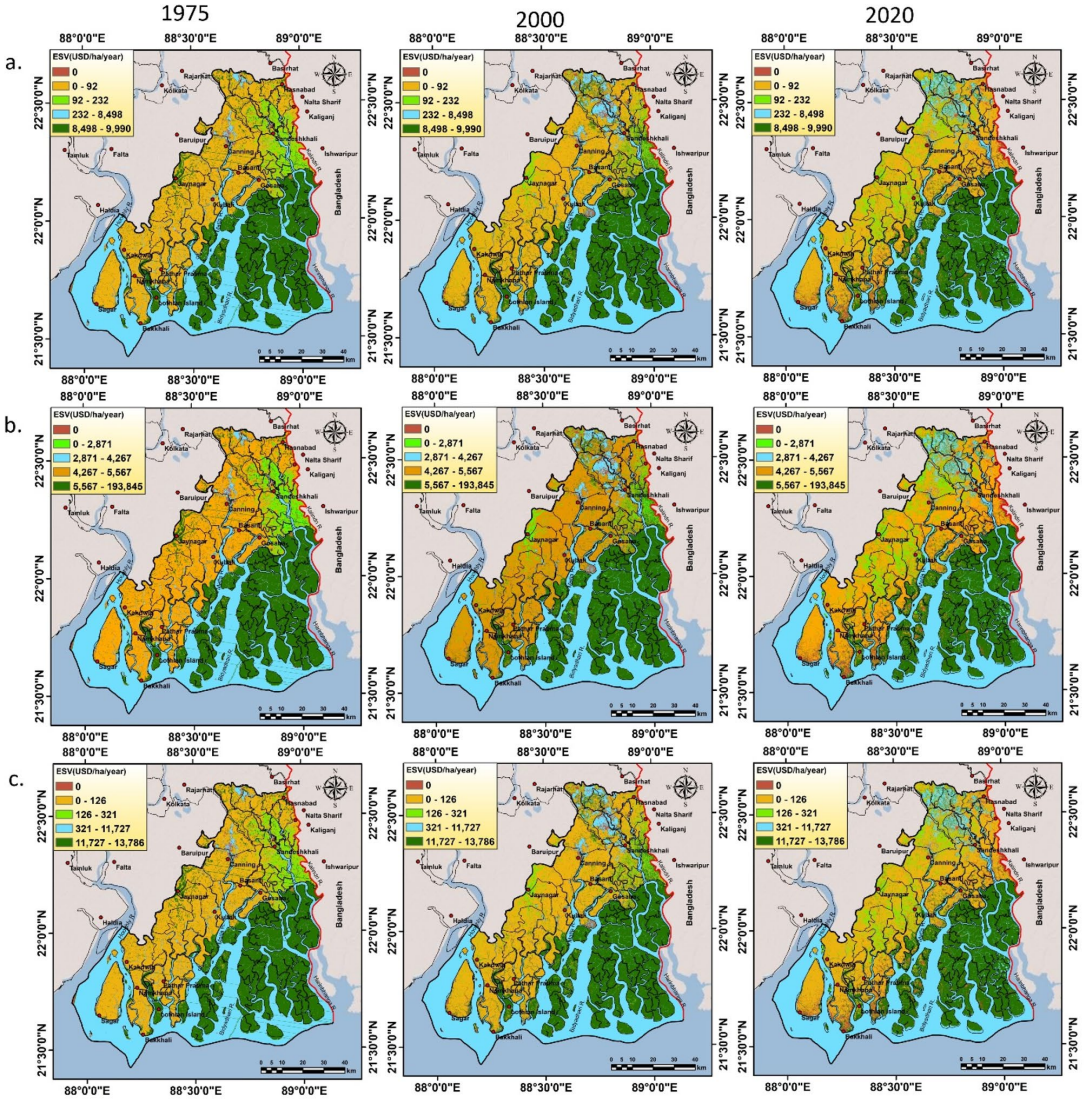
Spatial ordination of ecosystem service values (ESVs) (USD/ha/year) in 1975, 2000 and 2020 within the entire study region using (**a**). C97a (**b**). D12 (**c**). C97b unit values. The maps were prepared using ArcGIS 10.3 software [https://desktop.arcgis.com].

For estimation of the perfect ESVs of different ecosystem units in the Sundarban biosphere reserve, several ecosystem valuation unit values, i.e., Costanza et al.^1^, Costanza et al.^4^ (C97a, C97b, C11), De Groot et al.^25^ (D12), Xie et al.^29^ (X8) have been employed as a value coefficient (Table S4 and Fig. 3). Many scientific research articles on standard monetary values for different ecosystem services are the primary tool to carry out the whole research process^1,4,25,29^. The value of the ESs shows a relative contribution of various ecosystem components. So, it needs to include different methods to assess the benefits of individual nature’s services. There are some existing challenges to measure the actual valuation of ESs. Some popular valuation methods like avoided and replacement cost estimates are not depended on every ones perceptions^4^. The relative monetary contribution of ESs can be expressed in multiple units. In essence, any of the contributors to the production of benefits can be used as denominator and the others are expressed in terms of it^4^. The determined LULC classes of the referenced years (1975, 2000 and 2020) have been used as a proxy to estimate the precise ESVs of the individual LULC classes of the each referenced years (Fig. 2). The value coefficients are assigned by the above mentioned ecologists and different ecosystem services (Fig. 4) are multiplied with the area (ha) of the respective LULC class. The combined ecosystem service values of all land use land cover classes represent as total ecosystem service value (TESV) for the explorative year.

It follows,

2 $$ ESV_{i} = \left( {A_{k} *VC_{k} } \right) $$

3 $$ ESV_{t} = \Sigma \left( {A_{k} *VC_{k} } \right) $$

Here, ${ESV}_{i}$ & ${ESV}_{t}$ indicate the ecosystem service values (ESVs) of individual and total LULC classes respectively. $A$ signifies area in hectare and $VC$ represents value coefficient $(USD {ha}^{-1}{year}^{-1})$.

Functional diversity of the plant defines the range, value and relative abundance of plants in a particular ecosystem^30^ and it is one of the key controllers of various ecosystem services including carbon sequestration, soil nutrient retention, pollination etc.^30^. A recent scientific study shows that carbon stock (an important ecosystem service) has been significantly determined in mangrove regions using various structural parameters, such as wood specific gravity, total weight, circumference of the tree, stem and quantity of leaf and branches etc.^31^.

Quantification of natural goods and services replicates the uncertainties in biophysical sciences. Uncertainties refer the variability among the ecosystem services. However, a detail understanding about the uncertainties among the ecosystem services is more essential for the better valuation of ecosystem services^32^. The interrelationship among the ecosystem services and its valuation means that the valuation or value of any ecosystem services depends on its interrelationship with the other services. The changing pattern of any services has a significant effect on other ecosystem services and valuation^32^.

### The changing trend determination of ecosystem services in the various years

The changing patterns of ecosystem services (CES) in various years have been measured by different ecosystem value change indices for each LULC type (x). The ecosystem service values (ESVs) have been measured by USD (United State Dollar). Different change indices follow,

4 $$ CES_{x} = \left[ {\frac{{ESV_{{\left( {n + 1 year} \right)x}} - ESV_{{\left( {n \;year} \right)x}} }}{{ESV_{{\left( {n \;year} \right)x}} }}} \right] \times 100 $$

Here, n indicates referenced year whereas n + 1 represents the next referenced year.

Similarly, the change percentage of ecosystem service values has been detected by the following formula,

5 $$ ESV\; change \left( \% \right) = \left[ {\frac{{ESV_{final \;year} - ESV_{initial \;year} }}{{ESV_{initial \;year} }}} \right] \times 100 $$

For the estimation of the impact of LULC change over ecosystem service values (ESVs), the coefficient of sensitivity analysis (CS) has been performed here. The coefficient of sensitivity (CS) of ecosystem service values relation with LULC change has been determined using the concept of elasticity. The concept of CS is totally based on standard economic concept of elasticity. It follows as,

6 $$ CS = \frac{{\left( {ESV_{J} - ESV_{i} } \right)/ESV_{i} }}{{\left( {VC_{jk} - VC_{ik} } \right)/VC_{ik} }} $$

Here, ESV signifies the ecosystem service values of the eco-regions and VC represents the value coefficient. $i and j$ follow the initial and adjusted values respectively whereas $k$ is the LULC types. The static property and elastic properties are depending on VC. Meanwhile, if the changes of ecosystem service values and value coefficient exceed the threshold limit (> 1) then estimated ecosystem service value is elastic respect with value coefficient, and it will be totally inelastic when the value is < 1^33,34^.

### Application of InVEST carbon model for carbon storage estimation

The Integrated Valuation of Ecosystem Services and Trade-offs (InVEST) model is a branch of models that were developed by Natural Capital Project collaborated with 250 groups of organizations such as WWF (world wildlife fund), Stanford University, TNC (the nature conservancy) etc.^35^. The InVEST-carbon model can explicitly determine the static carbon stock over a study region^36^. The InVEST-carbon model follows the natural carbon cycle and it ultimately determines the stored amount of static carbon by aggregating values of carbon pools (aboveground biomass, belowground biomass, dead organic matter and soil organic carbon) of various ecosystem units^37^. For estimation of the carbon density of each grid cells, the LULC map has been used as a primary input. The carbon pool parameters have been taken from various researches^35,38,39^, forest survey report and IPCC guidelines (2006)^40^. The InVEST carbon model considers the basic carbon cycle concept, the total amount of carbon storage (static) and sequestration of the carbon amount based on the above mentioned carbon pool as well as storage data^41^. The carbon density of each land use land cover type (from carbon pool table) has been used as a primary data to estimate the carbon storage of each grid cell^42^. Finally, the total amount of carbon storage has been determined through aggregation of the carbon pool values that are assigned for each LULC types^37^. Carbon pool data (AGB, BGB and SOC) of mangrove forest areas have been determined by a primary survey of twelve different selected locations within SBR. A detailed primary survey was conducted in December, 2019 to investigate the carbon pool data (AGB (above ground biomass), BGB (below ground biomass) and organic soil) within the different pockets of SBR region. Total 12 areas have been selected based on the abundance of mangrove forest (Table S1). For measure the above ground biomass of the sample sites, total number of branches and its corresponding circumferences have been measured. Circumferences of branches have been categorized into different classes based on basal diameter. Leaves had been removed and dried it in laboratory (wood specific gravity) to remove the moisture contents within the branches. Then the weight of the branches from separate groups has been registered and calculates the AGB. The above ground leaf and stem biomass have been estimated through the measurement of the weight of total leaf and stems after oven dried condition. Tree roots were measured for the determination of below ground biomass. Soil samples were taken by removing the top soil (15–30 cm below from the surface). According to the InVEST model, the static carbon density of individual land use land cover type can be determined by the following equation,

7 $$ C_{i} = C_{{i\left( {above} \right)}} + C_{{i\left( {below} \right)}} + C_{{i\left( {dead} \right)}} + C_{{i\left( {soil} \right)}} $$

Here,${C}_{i(above)}$, ${C}_{i(below)}$, ${C}_{i(dead)}$ and ${C}_{i\left(soil\right)}$ indicate aboveground carbon density, belowground carbon density, dead organic material and soil organic material carbon density respectively. However the total carbon storage of the area can be determined by the following formula,

8 $$ C_{total} = \mathop \sum \limits_{i}^{n} C_{i} *A_{i} $$

Here, ${C}_{total}$ signifies the total carbon storage in the study area. ${A}_{i}$ indicates the area of individual classes.

Sundarban region is the store house of blue carbon^21^. ‘Blue carbon’ refers the carbon that is captured by various organisms and stored into the coastal sediments including salt marshes, sea grasses, mangroves etc.^43^. Chowdhury et al.^21^ concluded that plantation of ‘Avicennia marina’ using ‘direct propagule dibbling’ method can be sequestered the maximum volume of blue carbon. The InVEST carbon biophysical approach has been used to investigate the carbon pool and carbon sequestration in SBR region based on grid cell value. Blue carbon fluxes and pool have been excluded in this study. InVEST model has been widely used all over the world to determine the carbon sequestration value based on spatial resolution. A previous research also shows the gradual decreasing rate of carbon storage in SBR region based on InVEST model (2003–2013)^44^.

### ESVs change hotspot analysis

A hotspot represents a region with high concentration of a particular matter in a restricted place. In this research, ecosystem service values convexity and detraction indicate the areas where high change of ESVs (positive and negative) has been occurred. For analysis the ESVs change hotspots (convexity and detraction) within the region, Getis-Ord Gi* method^45^ has been applied in ArcGIS 10.3 environment using 3 unit values (C97a, D12 and C97b). Gi* statistic indicates a Z-score value which can reflect the intensity of clustering^46^. Statistically significant positive and negative GiZ value (*p* < 0.05) indicates hotspot (convexity areas of ESVs) and cold spot (detraction areas of ESVs) respectively. Ultimately the inverse distance weightage (IDW) method has been applied for clear demarcation of hotspot and cold spot region under SBR within the time frame on the basis of GiZ value. It follows as,

9 $$ GI^{*} = \frac{{\mathop \sum \nolimits_{j = 1}^{n} w_{ij} x_{j } - \overline{X}\mathop \sum \nolimits_{j = 1}^{n} w_{i,j} }}{{S\sqrt {\frac{{[n\mathop \sum \nolimits_{j = 1}^{n} w^{2}_{ij} - (\mathop \sum \nolimits_{j = 1}^{n} w_{i,j} )]}}{n - 1}} }} $$

${x}_{j}$, and ${w}_{i,j}$ represent j features attribute value and spatial weight among the feature I and j respectively whereas n signifies total number.

To calculate value $\overline{\mathrm{X} }$ and S, it follows as

10 $$ \overline{X} = \frac{{\mathop \sum \nolimits_{j = 1}^{n} x_{j } }}{n} $$

11 $$ S = \sqrt {\frac{{\mathop \sum \nolimits_{j = 1}^{n} x^{2}_{j } }}{n} - (\overline{X}} ) $$

## Result

### Analysis of the spatiotemporal dynamic pattern of LULC

Detailed description of LULC change statistics has been illustrated in Table 1. The LULC table of 1975 shows that cropland (34.48%) had the highest share of area in SBR region, followed by water body including rivers and creeks (31.12%), mangrove forest (29.74%). In 2000, water bodies including rivers and creeks (33.80%) had the highest share of area, followed by croplands (31.84%), mangrove forest (28.54%). Whereas in 2020, water body including rivers, creeks (35.38%) had the highest share of area, followed by croplands (30.42%) and mangrove forest (25.19%) (Table 1). It can be easily concluded that the entire SBR region is primarily occupied by water bodies, croplands and mangrove forest within the research period. The drastic gain of build-up area or settlement has been observed within 1975–2020 (7.77% per year). The net area of every classes has been reduced due to infrastructural development (roads, buildings, houses etc.), followed by sparse forest or open forest (0.84% per year) and water body (0.30% per year). Croplands (0.26% per year), mangrove forest (0.34% per year) and fallow land (0.14% per year) have been pursued a negative trend within the period 1975 to 2020. Error statistics or error matrix shows that the accuracy of the land use land cover maps is 84.4%, 86% and 86.8% for the year 1975, 2000 and 2020 respectively (Table S6).

**Table 1:** Changing pattern of LULC within the study period.

LULC classes	1975		2000		2020		1975–2000		2000–2020		1975–2020	
	Area (ha)	%	Area (ha)	%	Area (ha)	%	Δ Area (ha)	% change year^-1^	Δ Area (ha)	% change year^-1^	Δ Area (ha)	% change year^-1^
Waterbody	297,775	31.12	323,407	33.80	338,554	35.38	25,632	0.34	15,147	0.23	40,779	0.30
Settlement/build-up area	8166	0.85	23,669	2.47	36,715	3.84	15,503	7.59	13,046	2.76	28,549	7.77
Croplands	329,918	34.48	304,623	31.84	291,058	30.42	− 25,295	− 0.31	− 13,565	− 0.22	− 38,860	− 0.26
Mangrove forest	284,597	29.74	273,061	28.54	241,053	25.19	− 11,536.4	− 0.16	− 32,008	− 0.59	− 43,544	− 0.34
Sparse forest/open forest	34,995	3.66	31,294	3.27	48,165	5.03	− 3700.72	− 0.42	16,871	2.70	13,170	0.84
Fallow land	1425	0.15	824	0.09	1333	0.14	− 601.44	− 1.69	509	3.09	− 92	− 0.14

### States of estimated ecosystem service values

The issues regarding eco-system service valuation have been raised mainly from the decisions and choices. Some researchers argued that valuation of ecosystem services is not accurately possible^1^. Valuation means measuring the trade-offs towards attaining a goal^47^. The valuation of ecosystem services is therefore a relative contribution of ecosystem on that goal^4^. Presently, we have multiple ways to assess the contribution of ecosystem services and some based on individual perceptions. ‘Willingness-to-pay’ of the individuals towards ecosystem services either directly or indirectly is a significant method for estimation the value of ecosystem services^1^.

The total ecosystem service values of the entire Sundarban Biosphere Reserve (SBR) are 27,450.42, 26,665.99 and 24,139.63 million USD for the year 1975, 2000 and 2020 respectively (Table 2). Estimated total loss of ecosystem service value is 3310.79 million USD within the research period (1975–2020). At the commencement of the study period (1975), water body (including river/creeks), settlement/build-up areas, cropland, mangrove forest, sparse forest or open forest have been accounted 2702.72 (9.83%) (1270.61–3725.77), 54.36 (0.20%) (0–54.36), 845.34 (3.07%) (30.35–1836.65), 23,823.26 (86.65%) (2014.71–55,167.79) and 68.21(0.25%) (8.12–145.79) million USD ecosystem service values respectively (Table S5). In 2000, the same LULC classes have been accounted 2935.36 (10.96%) (1379.98–4046.47), 157.66 (0.59%) (0–157.66), 780.53 (2.91%) (28.03–1695.84), 22,857.56 (85.31%) (1933.05–52,930.96) and 60.99 (0.23%) (7.26–130.37), million USD ecosystem service values respectively. Similarly, in 2020, the same LULC classes have been accounted 3072.84 (12.63%) (1444.61–4235.99), 244.56 (1.00%) (0–244.56), 745.77 (3.06%) (26.78–1620.32), 20,178.22 (82.92%) (1706.46–46,726.92) and 93.88(0.39%) (11.17–200.66), million USD ecosystem service values respectively (Table 2). Croplands and sparse or open forest region have contributed a minimum proportion of ecosystem service values in both the years. Mangrove forest also accounts highest proportion in ESVs, 86.65%, 85.31% and 82.92% for the year 1975, 2000 and 2020 respectively and it follows a negative trend within the restricted time frame. Similarly, a positive trend of ESVs has also been observed in the case of water body including rivers and creeks (2702.72, 2935.36 and 3072.84 million USD in the year 1975, 2000 and 2020 respectively). It has been observed that mangrove forest region has the highest ecosystem service value (it is classified, 8498–9990 USD year^-1^ ha for C97a method, 5567–193,845 USD year^-1^ ha for D12 method and 11,727–13,786 USD year^-1^ ha for C97b method).

**Table 2:** Calculated ESVs (million USD yr^-1^) derived from five unit values within the period 1975–2020.

LULC classes	Unit values	Million USD ya^-1^					
		1975	%	2000	%	2020	%
Waterbody (lakes/rivers)	C97a	2530.50	46.76	2748.31	49.87	2877.03	54.05
	C97b	3492.01	46.76	3792.59	49.87	3970.22	54.05
	C11	3725.77	6.11	4046.47	6.86	4235.99	7.99
	D12	1270.61	2.18	1379.98	2.46	1444.61	2.89
	X8	2494.72	49.24	2709.46	52.56	2836.35	55.93
	Mean	2702.72		2935.36		3072.84	
Settlement/build-up area	C97a	0.00	0.00	0.00	0.00	0.00	0.00
	C97b	0.00	0.00	0.00	0.00	0.00	0.00
	C11	54.39	0.09	157.66	0.27	244.56	0.46
	D12	0.00	0.00	0.00	0.00	0.00	0.00
	X8	0.00	0.00	0.00	0.00	0.00	0.00
	Mean	54.36		157.66		244.56	
Cropland	C97a	30.35	0.56	28.03	0.51	26.78	0.50
	C97b	41.57	0.56	38.38	0.50	36.67	0.50
	C11	1836.65	3.01	1695.84	2.88	1620.32	3.06
	D12	1836.65	3.15	1695.84	3.02	1620.32	3.25
	X8	481.49	9.50	444.58	8.62	424.78	8.38
	Mean	845.34		780.53		745.77	
Mangrove forest	C97a	2843.13	52.53	2727.88	49.49	2408.12	45.24
	C97b	3923.46	52.54	3764.42	49.50	3323.16	45.24
	C11	55,167.22	90.54	52,930.96	89.77	46,726.44	88.12
	D12	55,167.79	94.51	52,931.51	94.36	46,726.92	93.58
	X8	2014.71	39.77	1933.05	37.50	1706.46	33.65
	Mean	23,823.26		22,857.56		20,178.22	
Sparse forest/open forest	C97a	8.12	0.15	7.26	0.13	11.17	0.21
	C97b	11.23	0.15	10.05	0.13	15.46	0.21
	C11	145.79	0.24	130.37	0.22	200.66	0.38
	D12	100.47	0.17	89.85	0.16	138.28	0.28
	X8	75.44	1.49	67.47	1.31	103.84	2.05
	Mean	68.21		61.00		93.88	
Fallow land	C97a	0.00	0.00	0.00	0.00	0.00	0.00
	C97b	0.00	0.00	0.00	0.00	0.00	0.00
	C11	0.00	0.00	0.00	0.00	0.00	0.00
	D12	0.00	0.00	0.00	0.00	0.00	0.00
	X8	0.00	0.00	0.00	0.00	0.00	0.00
	Mean	0.00		0.00		0.00	
Total value	C97a	5412.10		5511.48		5323.10	
	C97b	7468.28		7605.44		7345.51	
	C11	60,929.82		58,961.30		53,027.96	
	D12	58,375.52		56,097.17		49,930.13	
	X8	5066.37		5154.54		5071.43	
	Mean	27,450.42		26,665.99		24,139.63	

### Changes of ESVs values

The total ecosystem service values (TESV) in SBR region have been quantified in different referenced years. The average total ESVs within the SBR region are 27,450.42 (5066.37–60,929.82), 26,665.99 (5154.54–58,961.30) and 24,139.63 (5071.43–53,027.96) million USD for 1975, 2000 and 2020 respectively. The net loss of TESVs in 1975–2000 and 2000–2020 was around 784.43 and 2526.36 million USD respectively whereas the net loss of overall TESVs in the study period is 3310.79 million USD. In overall study period (1975–2020) the ESVs of mangrove forest class have been mostly suffered. The total net loss of ESVs in mangrove class is 3645.05 (15.30%) million USD, followed by croplands that is 99.57 (11.78%) million USD while the ESVs of water body, settlement, sparse forest have slightly increased i.e. 370.12 (13.69%), 190.2 (349.61%) and 25.67 (37.63%) million USD respectively. It has been observed that during the study period mangrove forest contributes most of the ESVs in the SBR area whereas settlement and sparse forest contribute small amount of ESVs. Considering the temporal periods (1975–2000 and 2000–2020), it has been observed that mangrove forest follows a continuous decreasing trend of ESVs, 965.7 (4.05%) (81.67–2236.28) and 2679.35 (11.72%) (226.59–6204.59) respectively followed by croplands, 64.81 (7.67%) (2.33–140.82) and 34.76 (4.45%) (1.25–75.52) respectively (Table 3). In the total study period (1975–2020), the valuation of all type of ES is diminished except provisional services. Total 121.47, 7.56 and 19.25 million USD is reduced for regulating, supporting and cultural services respectively whereas total 59.44 million USD of provisional services has increased within the time frame (1975–2020) (Table 5). The values of particular ecosystem services of each unit values are different but the ANOVA shows that the mean of valuation methods is not significantly different (*p* > 0.05). Here, the Chi-Square test shows that there is no statistical significant relationship among the unit values (*p* > 0.05).

**Table 3:** Estimated ESVs of the each LULC classes of different years and its temporal dynamics using the applied unit values respectively.

LULC	ESVs (million USD)	Changes										
	1975	2000	2020	1975–2000	%	% year^1^	2000–2020	%	% year^1^	1975–2020	%	% year^1^
Waterbody (lakes/rivers)	2530.50	2748.31	2877.03	217.82	8.61	0.34	128.72	4.68	0.23	346.54	13.69	0.30
	3492.01	3792.59	3970.22	300.58			177.63			478.21		
	3725.77	4046.47	4235.99	320.70			189.52			510.22		
	1270.61	1379.98	1444.61	109.37			64.63			174.00		
	2494.72	2709.46	2836.35	214.74			126.90			341.64		
				232.64			137.48			370.12		
Settlement/build-up area	0.00	0.00	0.00	0.00			0.00			0.00		
	0.00	0.00	0.00	0.00			0.00			0.00		
	54.39	157.66	244.56	103.27	189.85	7.59	86.90	55.12	2.76	190.17	349.61	7.77
	0.00	0.00	0.00	0.00			0.00			0.00		
	0.00	0.00	0.00	0.00			0.00			0.00		
				103.27			86.90			190.17		
Cropland	30.35	28.03	26.78	−2.33	−7.67	−0.31	−1.25	−4.45	−0.22	−3.58	−11.78	−0.26
	41.57	38.38	36.67	−3.19			−1.71			−4.90		
	1836.65	1695.84	1620.32	−140.82			−75.52			−216.33		
	1836.65	1695.84	1620.32	−140.82			−75.52			−216.33		
	481.49	444.58	424.78	−36.92			−19.80			−56.71		
				−64.81			−34.76			−99.57		
Mangrove forest	2843.13	2727.88	2408.12	−115.25	−4.05	−0.16	−319.76	−11.72	−0.59	−435.01	−15.30	−0.34
	3923.46	3764.42	3323.16	−159.04			−441.26			−600.30		
	55,167.22	52,930.96	46,726.44	−2236.26			−6204.53			−8440.78		
	55,167.79	52,931.51	46,726.92	−2236.28			−6204.59			−8440.87		
	2014.71	1933.05	1706.46	−81.67			−226.59			−308.26		
				−965.7			−2679.35			−3645.04		
Sparse forest/open forest	8.12	7.26	11.17	−0.86	−10.58	−0.42	3.91	53.91	2.70	3.06	37.64	0.84
	11.23	10.05	15.46	−1.19			5.42			4.23		
	145.79	130.37	200.66	−15.42			70.28			54.87		
	100.47	89.85	138.28	−10.62			48.44			37.81		
	75.44	67.47	103.84	−7.98			36.37			28.39		
				−7.21			32.88			25.67		
Fallow land	0.00	0.00	0.00	0.00			0.00			0.00		
	0.00	0.00	0.00	0.00			0.00			0.00		
	0.00	0.00	0.00	0.00			0.00			0.00		
	0.00	0.00	0.00	0.00			0.00			0.00		
	0.00	0.00	0.00	0.00			0.00			0.00		
				0			0			0		
Unit values	5412.10	5511.48	5323.10	99.38	1.84	0.07	−188.37	−3.42	−0.17	−88.99	−1.64	−0.04
	7468.28	7605.44	7345.51	137.16	1.84	0.07	−259.93	−3.42	−0.17	−122.76	−1.64	−0.04
	60,929.82	58,961.30	53,027.96	−1968.52	−3.23	−0.13	−5933.34	−10.06	−0.50	−7901.86	−12.97	−0.29
	58,375.52	56,097.17	49,930.13	−2278.35	−3.90	−0.16	−6167.04	−10.99	−0.55	−8445.39	−14.47	−0.32
	5066.37	5154.54	5071.43	88.17	1.74	0.07	−83.12	−1.61	−0.08	5.06	0.10	0.00
Mean	27,450.42	26,665.99	24,139.63	−784.43	−2.86	−0.11	−2526.36	−9.47	−0.47	−3310.79	−12.06	−0.27

### Assessment of carbon sequestration and analysis of hotspots

**Figure 5:**
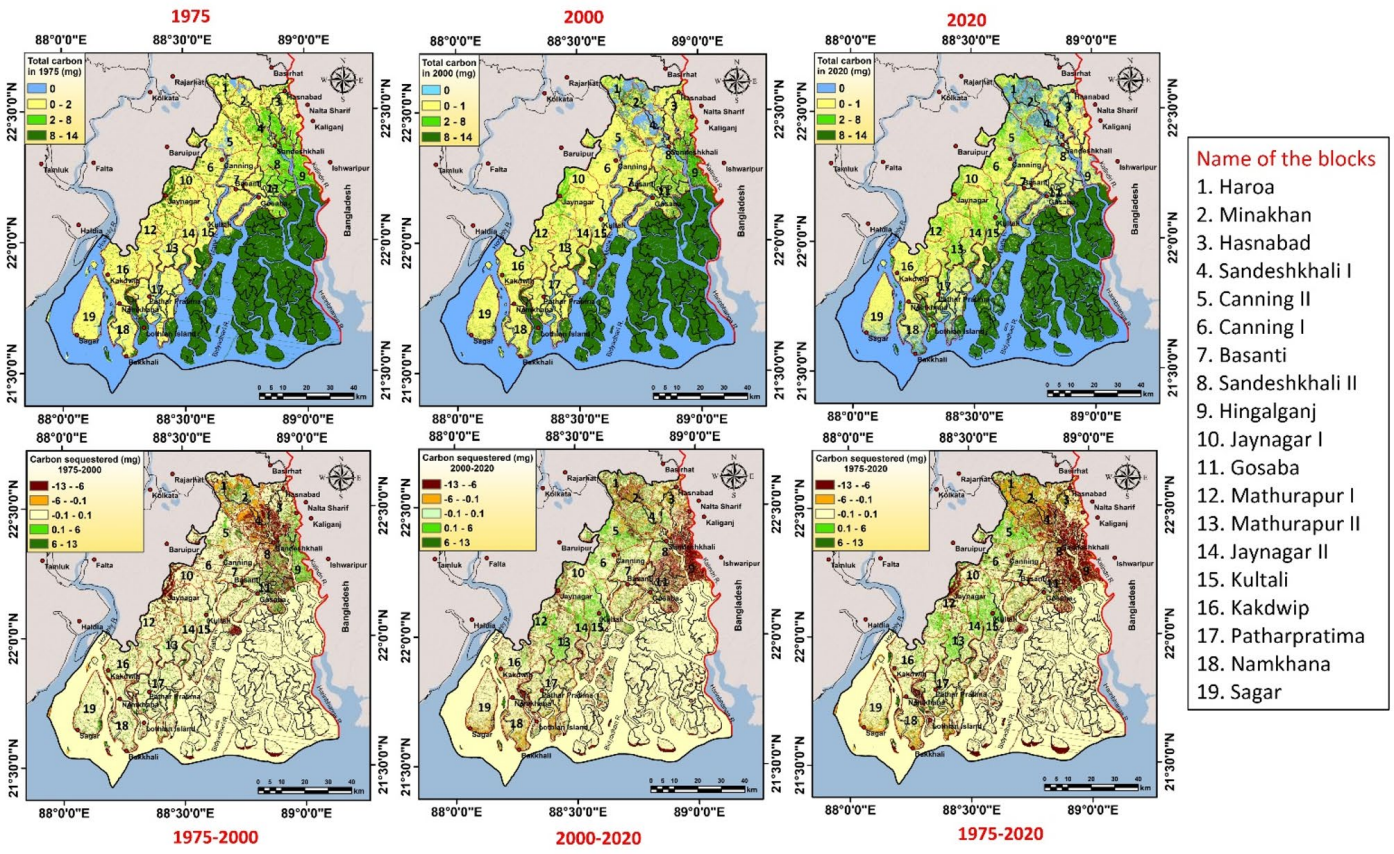
Total carbon stock amount (mg/ha) in the year 1975, 2000 and 2020 along with carbon sequestered from 1975 to 2000, 200 to 2020 and 1975 to 2020. The maps were prepared using ArcGIS 10.3 software [https://desktop.arcgis.com].

**Figure 6:**
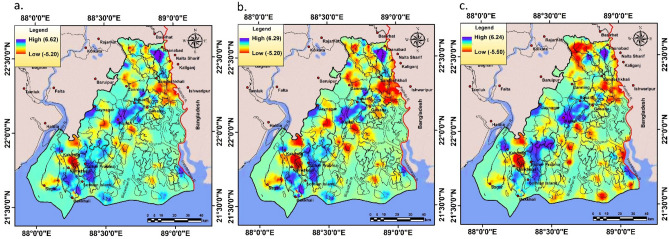
Identified changing ESVs hotspot and cold spot region in the study area within (1975–2020) using (**a**) C97a (**b**) D12 and (**c**) C97b unit values. The maps were prepared using ArcGIS 10.3 software [https://desktop.arcgis.com].

The InVEST- carbon storage and sequestration model has illustrated the result of spatiotemporal distribution of static carbon by the raster grid data over SBR region. The result of InVEST-carbon model has been revealed that the total carbon storage in SBR region is 48.87Tg, 46.65Tg and 43.33Tg for 1975, 2000 and 2020 respectively (Fig. 5). Vegetation or any type of forest areas is generally stored more carbon than other land use land cover type^48^. However croplands can take a significant contribution in total static carbon storage by capturing more soil carbon^35^. In 1975, the stored carbon ranges from 17,105 mg (fallow land) to 41,266,629 mg (mangrove forest) in SBR. In 2000, the stored carbon ranges from 9888 (fallow land) to 39,593,845 mg (mangrove forest) whereas in 2020, carbon store ranges from 15,996 mg (fallow land) to 34,952,685 mg (mangrove forest) (Table 4). Due to conversion and reduction of forest lands (mangrove forest area), capacity of carbon sequestration has been significantly reduced over the study region (Fig. 5). Total 5,537,566 mg loss of carbon stock has been determined in the study area within the period 1975–2020. A huge reduction of carbon storage has been observed over mangrove forest (6,313,944 mg), followed by agricultural land (544,039 mg) whereas sparse forest has revealed a positive storage of carbon pool (1,093,133 mg). The map shows that mangrove forest area contains highest amount of static carbon (8–14 mg of carbon in each grid). The region outside of mangrove area contains lower amount of static carbon (0–2 mg for settlement, waterbody and fallow land; 2–8 mg for sparse forest in each grid cell). The significant carbon sequestered blocks under SBR region are Mathurapur II, Jaynagar II, Kultali whereas Haroa, Minakhan, Hasnabad, Sandeshkhali I, Gosaba, Hingalganj, Namkahan have faced a significant problem of carbon loss. Total 138.4 km^2^ mangrove areas have been destructed in Indian part of Sundarban in last 30 years. Around 57% total mangroves have been lost due to erosion and 22% area had been converted into fallow land while remaining part was transformed into anthropogenic use. The high tidal fluctuation during the cyclone Aila heavily destructed the coast of Indian Sundarban and decreases the mangrove cover and as a result, the blue carbon storage has been depleted in this area^49^. For the accurate demarcation of hotspot (convexity/gain of ESVs) and cold spot (detraction/loss of ESVs) areas of ecosystem service values (Using C97a, C97b and D12 unit values), Getis-Ord Gi^*^ statistic has been applied (Fig. 6). Figure 6 shows that some parts of Kultali, Namkhana, Sagar, Patharpratima, Hasnabad are ESVs hotspot region (convexity of ESVs) and some parts of Patharpratima, Hingalganj, Sandeshkhali, Gosaba, Kakdwip are under the cold spot (detraction of ESVs) region that indicates a high devaluation of ecosystem service values. In 1975, total provisional, regulating, supporting and cultural services were 841.49, 4261.58, 53.63 and 255.82 million USD yr^-1^. In 2000, the total value of these services was 887.94, 4318.57, 51.23 and 254.12 82 million USD yr^-1^ respectively whereas in 2020, these were 900.93, 4140.11, 46.07 and 236.58 million USD yr^-1^ respectively (according to Costanza et al.^1^ (C97a) unit value) (Table 5).

**Table 4:** Total amount of stored carbon along with change dynamic in SBR region.

Proxy biomes	Carbon stock (mg)			Changes (mg)		
	1975	2000	2020	1975–2000	2000–2020	1975–2020
Waterbody	0	0	0	0	0	0
Settlement/Build-up area	65,327	189,352	293,720	124,025	104,368	228,393
Cropland	4,618,851	4,264,722	4,074,812	−354,129	−189,910	−544,039
Mangrove forest	41,266,629	39,593,845	34,952,685	−1,672,784	−4,641,160	−6,313,944
Sparse forest/open forest	2,904,562	2,597,402	3,997,695	−307,160	1,400,293	1,093,133
Fallow land	17,105	9888	15,996	−7217.28	6108	−1109.28
Total	48,872,474	46,655,209	43,334,908

**Table 5:** ESVs of different services along with its change dynamics.

Ecosystem services	Ecosystem service values (million USD /ha/year)			Changes		
	1975	2000	2020	1975–2000	2000–2020	1975–2020
Provisional service	841.49	887.94	900.93	46.45	12.98	59.44
Regulating services	4261.58	4318.57	4140.11	56.99	−178.45	−121.47
Supporting services	53.63	51.23	46.07	−2.40	−5.16	−7.56
Cultural services	255.82	254.12	236.58	−1.70	−17.54	−19.25

### Impact of the changes of LULC over ESVs

The impact of the changing pattern of LULC over ESVs notably observed by the area contribution of each LULC type and it respected ESVs within the time frame. Generally, in this research period (1975–2020), the area of agricultural land and mangrove forest is consistently diminished. Total areas of different mangrove categories (Phoenix, Avicennia, mangrove scrub, mixed mangrove etc.) have together shared 284,597 ha, 273,061 ha and 241,053 ha for the years 1975, 2000 and 2020 respectively and it is revealed that total 43,544 ha areas of mangrove had been loosed in the whole study period (1975–2020). The area of mangrove forest was 29.74% in 1975 and 25.19% in 2020. Similarly, cropland has been reduced its area from 34.48% in 1975 to 30.42% in 2020 whereas the area of water body has been increased 31.12% in 1975 to 35.38% in 2020. For instance, ecosystem service values of mangrove forest decreased from 86.65% in 1975 to 82.92% in 2020. Similarly, the ESVs of cropland dropped 3.07% in 1975 to 3.06% in 2020.The ESVs of wetlands have been increased from 9.83% in 1975 to 12.63% in 2020. Generally, it can be concluded that changes of natural forest ecosystem can significantly affect the ESVs over SBR. So, the changes of LULC have a significant effect over the value of ecosystem services.

### Sensitivity analysis of ecosystem services

The reliability of the used value coefficients (VC) for each eco-region or proxy biome (equivalent biome which has uniformity with the selected LULC classes) has been validated using coefficient of sensitivity (CS). It has been observed that the CS values are less than 1 in all cases that means the estimated ESVs are relatively inelastic respect with land use land cover categories^50^. The result indicates that water body (0.02–0.49) and mangrove forest (0.40–0.95) are more sensitive than other land alterations in 1975. The resembling trend has been observed in the case of 2000 (0.02–0.53 and 0.38–0.94 for water body and mangrove forest respectively) and 2020 (0.03–0.56 and 0.34–0.94 for water body and mangrove forest respectively) (Table 6). Other classes contribute very low CS values. Relatively low Coefficient of severity value reflects that the area of LULC and respected value coefficient is relatively small and vice versa^50^. The aggregate result significantly shows that water body including rivers and creeks and mangrove forest are more sensitive. This recognition has resisted the protection of water bodies, river networks and mangrove ecological unit due to its richness of ESVs.

**Table 6:** Coefficient of sensitivity (CS) of ESVs respected with LULC changes for the reference years using five various unit values.

LULC	Unit values	1975	2000	2020
Waterbody	C97a	0.47	0.50	0.54
	C97b	0.47	0.50	0.54
	C11	0.06	0.07	0.08
	D12	0.02	0.02	0.03
	X8	0.49	0.53	0.56
	Mean	0.30	0.32	0.35
Settlement	C97a	0.00	0.00	0.00
	C97b	0.00	0.00	0.00
	C11	0.00	0.00	0.00
	D12	0.00	0.00	0.00
	X8	0.00	0.00	0.00
	Mean	0.00	0.00	0.00
Croplands	C97a	0.01	0.01	0.01
	C97b	0.01	0.01	0.00
	C11	0.03	0.03	0.03
	D12	0.03	0.03	0.03
	X8	0.10	0.09	0.08
	Mean	0.03	0.03	0.03
Mangrove forest	C97a	0.53	0.49	0.45
	C97b	0.53	0.49	0.45
	C11	0.91	0.90	0.88
	D12	0.95	0.94	0.94
	X8	0.40	0.38	0.34
	Mean	0.66	0.64	0.61
Sparse forest/open forest	C97a	0.00	0.00	0.00
	C97b	0.00	0.00	0.00
	C11	0.00	0.00	0.00
	D12	0.00	0.00	0.00
	X8	0.01	0.01	0.02
	Mean	0.00	0.00	0.01
Fallow land	C97a	0.00	0.00	0.00
	C97b	0.00	0.00	0.00
	C11	0.00	0.00	0.00
	D12	0.00	0.00	0.00
	X8	0.00	0.00	0.00
	Mean	0.00	0.00	0.00

## Discussion

Presently, determination of ESVs and their dynamic pattern is totally based on proxy data analysis^4,51^. The accuracy of the valuation has been associated with the accuracy level of the land use land cover maps^52^. The open mangrove ecosystem was affected through the transformation of mangrove areas into croplands and the increasing demand of aquaculture during the period 1975 to 2006^53^. Due to the increasing trend of human intervention and its associated activities, total 3310.79 million USD losses have been detected over this region. Among the other land use land cover class, mangrove areas have the highest ecosystem service value (20,178.22 USD in 2020). In Sundarban area, mangrove forest and waterbody (both fresh and marshy water) are the primary producer of ecosystem services and values. Presently, due to human intervention and drastic LULC changes, ecosystem services are decreasing gradually^15^. Settlement or build up areas have the lowest ESVs (54.36 USD and 244.56 USD in 1975 and 2020).

The last few decades, the Sundarban mangrove ecosystem is being suffered by different natural and human induced adversity. The natural adversities include increasing salinity, sea level rise, coastal erosion, devastating cyclonic storms, river decay (disappearance of the river courses or beheaded from the principal rivers due to various natural and anthropogenic factors and these river courses were also the supplier of sweet water with sediments for the growth of mangroves.) and insufficient supply of sediments and freshwater, coastal flooding, embankment failure etc.^54^. The Sundarban mangrove ecosystem provides bulk of ESs such as food, fuel and freshwater (wood, honey, fish, crab, dung, tannin, leaves and twigs), fiber (timber, grasses, silk and wool), pharmaceuticals and biochemical (food and medicines), ornamental components (sells, skins and flowers), genetic resources (genes and genetic products) and cultural services (tourism, spiritual benefits and recreation) to exactly 20 million people in this region^55^. The primary ecological functions of SBR are hatching, breeding, spawning and nursing for pelagic and marine species.

In the recent years, the salinity is being increased in the eastern part of Indian Sundarban due to significant reduction of freshwater supply through the river systems like Mathabhanga-Churni-Ichhamati-Raimangal-Harinbhanga, Jamunna-Padma and Suti-Nona-Noai-Bidyadhari-Kultigang-Matla. Simultaneously, the ecological functions like hatching, breeding, spawning and nursing for pelagic and marine species in the SBR are being considerably reduced due to paucity of sediments or nutrients. The Sundarban mangrove is situated in the confluence of Ganges–Brahmaputra-Meghna river system or GBM delta. The Indian Sundarban is bounded by Dampier-Hodges line in the north, river Hugli in the west, roughly Ichhamati-Raimangal-Harinbhanga river system of Indo-Bangladesh international boundary in the east and Bay of Bengal in the south. Up to the end of fifteenth century, Bhagirathi-Hugli river system was the main flow of river Ganga after that it was started to divert towards the river Padma^56^. The river Bhagirathi-Hugli, Mathabhanga-Churni-Ichhamati-Raimangal-Harinbhanga, Jamunna-Padma, Suti-Nona-Noai-Bidyadhari-Kultigang-Matla and Adi Ganga and western tributaries of Bhagirathi-Hugli are the principal river networks of delta builders of Indian Sundarban. Most of the rivers have been disconnected from their parent rivers. The freshwater supplies along with sediments or nutrients have also been drastically reduced in the Sundarban region due to beheaded of off-take points of these rivers^57^. As a result the growth of mangroves in Indian Sundarban is being reduced in the recent years. The Bhagirathi-Hugli river system has been slightly rejuvenated after the construction of Farakka Barrage and the subsequent feeder canal in the year 1975. Similarly, the anthropogenic interferences within the river valley are highly responsible for degeneration of the fluvial system in this dynamic Ganga Delta region^22^. Here, the majority of people spend marginalized and vulnerable life and using forest as a livelihood subsistence support^22^. The impact of channelization like construction of bridge, culvert and sluice gate across the river channels has also been accelerated to decay the river system in the Sundarban Delta region.

The changes of mean sea level have been evidenced in the last few decades. Sagar Island station of Sundarban shows a substantial rising trend of sea level at a rate of 2.6–4 mm year^-1^ from 1985 to 2010^58^ as a result Sundarban mangrove ecosystem is going to submerge under the sea level. Consequently, the deltaic islands of Sundarban are being gradually submerged below the water level and large scale mangrove forests will be disappeared from this deltaic environment. An increasing trend of surface temperature (0.0119–0.045°c year^-1^) has been observed during last century around SBR and its surrounding area that will increase the sea level from 3.14 to 8 mm year^-1^ ^59^ and also destructs the mangrove forests^60^. The dynamics of landform buildings, formation of creeks and estuary have been partially disturbed due to disproportion of erosion and deposition, deficiency of sediments and freshwater supply, gradual sea level rise and increasing surface temperature etc.^22^. The nutrient input and salinity conditions determine the nutrient availability in the Sundarban estuary. The abundance of phytoplankton in this area has been totally controlled by the temperature, salinity and solar irradiance^61^. A recent study shows that rapid increase of greenhouse emission has been occurred in Sundarban region (methane (CH_4_), carbon dioxide (CO_2_), and nitrous oxide (N_2_O)) and a significant relation between water pollution and greenhouse gas emission has been determined^62^.

Frequent destructions of mangrove forests were evidenced due to devastating cyclones like Aila in 2009, Amphan in 2020 and Yaas in 2021 and the recurrent tropical cyclones continuously damage the Sundarban coast and significantly reduced the provisional, regulatory, supporting and cultural ecosystem services (Fig. 4) of Sundarban mangrove ecosystem^15^ (Table S3). Donato et al.^17^ revealed that mangrove forests are the most productive forest type and can store the significant amount of carbon (49 – 98%). He also showed that 0.02 to 0.12 Pg carbons have been released by the deforestation of mangrove forests. Furthermore, the mangrove ecosystem is very much efficient in fixing (15–46 × 10^12^ mol yr^-1^) and storing (3 × 10^14^ mol yr^-1^) carbon^63,64^ whereas the Sundarban mangrove ecosystem is only sequester roughly 25 × 10^10^ mol yr^-1^ of atmospheric co_2_^65^. Here, this study focused that the carbon storing capacity has been significantly reduced due to sequential reduction of mangrove forests in the territory of Indian Sundarban. The Sundarban mangrove forests are sequestering a large quantity of gaseous carbon; thus the protection and preservation of Sundarban mangrove ecosystem will be effectual environmental management strategies for the safeguarding of carbon balance and reduction of carbon dioxide emissions in the Sundarban deltaic environment^49,62^. Mangrove ecosystem acts as a highest carbon sequester and result of their declining trend is the potential greenhouse gas emission in the atmosphere^66^. A recent study shows that a significant variation has been observed among the various mangrove vegetation types^14^. The reasons behind the variation of carbon among vegetation types are the structure and above ground biomass pattern of the tree, size and basal area^67^. The carbon stock in SRB has been drastically changed within the study period. However, tree density forms a strong indicator of carbon stock^68^. Recently, drastic land use change and rapid modification of forest area can significantly decrease the vegetation cover as well as carbon stock in this ecosystem rich forest cover area^22,69^.

These climatic extremes accelerate the livelihood threats to billions of people who reside in the coastal stretch of India, Bangladesh, Myanmar, Thailand, Malaysia, China and other coastal countries of the world^70–72^. The large scale mangrove deforestation had occurred before and after independence mostly in the blocks of Sundarban like Sagar, Gosaba, Patharpratima, Basanti, Namkhana and Kultali (hot spots). The disappearance of mangrove is quicker than the other forests on the earth^73^. This study identified that there is a close nexus between development factors and mangrove ecosystem services degradation in Sundarban region. Rapid conversion of mangrove to cropland and brackish water aquaculture has been occurred due to high demand as well as profit mostly in different blocks of Basanti, Kultali and Gosaba etc.

Therefore, exact estimation of ecosystem service valuation and mapping is a significant robust tool for assessing the ecological as well as economic balance within a particular region or a specific ecosystem and this can help to develop policies against overexploitation of natural resources. The carbon storage process by InVEST model (bio-physical model) is depended on variation rate of LULC^74^. This short summary replicates us that any land management policy and strategies which are related with conversion of natural productive land into economic beneficial zones can overturn the relation of man and ecosystem balance^74^. In order to overwhelm the transitional problem between the taxonomic classification systems of ecosystem services, the Common International Classification of Ecosystem Services (CICES) was proposed in the year 2009 after that it was revised in the year 2013. It applies the three major sections of ecosystem services; regulatory, provisional and cultural ecosystem services. CICES did not include supporting services to avoiding the double counting during ecosystem services valuation; though it does not mean that supporting services have less important^75^.

## Conclusion

The concept of ecosystem services, valuation of ecosystem services, flow of ecosystem services and natural stock capital are significantly growing to measure the correlation and intensity of interdependency between human and nature. These concepts are similar to the concept of nature conservation with empirical valuation and measurement. The whole study gives us a clear illustration that ecosystem services have an important role and contribution in human welfare. We must give significant efforts towards the preservation of these natural capital stocks otherwise the human society or civilization will be fallen in great danger in near future. In this empirical research a detailed investigation has been done regarding the changes of ESVs in last 45 years by undertaking the dynamic pattern of land use land cover and corresponding value coefficients. Conversion of the ecosystem service values into monetary values does not mean that they are used as private commodities because many ecosystems produce goods as public assets. Some ecologists argued that methods of ESVs determination are totally meaningless, because if we loss or destruct all ecosystem services then the human civilization would be end, so the value must be endless^76^. Some researchers also claimed that proper estimation of ESV is impossible while all values are estimated mainly through the method of ‘willingness-to-pay’ that cannot surpass the aggregate of ability-to-pay. These types of estimations are based on virtual monetary value not real value and total expenditure should not be exceeded but it is very much important for determination of policies based on its loss/gain^77^. Authors think that new ecosystem service valuation should make considering regional demand of goods and services, nature of livelihood of people and functionality of mangrove ecosystem. There is a real research gap in this context and researchers will get multiple research wings to bring high precision research regarding the technique of ecosystem service valuation because universal valuation techniques would not clearly convey the local or regional scenario. Our investigation concluded that the quality of nature stored capitals was considerably dropped in the past years (27,450.42, 26,665.99 and 24,139.63 million USD for 1975, 2000 and 2020 respectively). The resulted drop of ecosystem services mainly related with the conversion of forest particularly mangrove forest land into other anthropogenic use. The high ecosystem values are concentrated over mangrove forest and water bodies because these eco-regions provide nonstop valuable good and services for human wellbeing. InVEST model also revealed that the total static carbon storage over the study area was 48.87, 46.65 and 43.33 Tg for the year 1975, 2000 and 2020 respectively. Total 6.31Tg loss of carbon has been observed in the case of mangrove forest during the overall study period (1975–2020). The final outcome of this study also helps in decision making towards sustainable utilization of natural resources. The sensitivity determination of ESVs and LULC can be a solution to the policymakers, administrator and environmentalist for adopting a suitable land use management over this eco-sensitive region. This natural capital will be more precious and scare in near future so this research will try to bring the proper valuation and assessment methods for this resource^1^. There is a huge future scope and research gap to discover new ecosystem valuation method as well as fundamental methodological layout for the conservation of valuable natural capital like Sundarban (mangrove ecosystem).

## Supplementary Information

Supplementary Information.

## Data Availability

The analysed data that support the findings of this study are available within the article and its Supplementary Information files.
