# Supplementary material for: Significant reduction of carbon stocks and changes of ecosystem service valuation of Indian Sundarban
Source: Sci Rep. 2022 May 12;12:7809. doi: 10.1038/s41598-022-11716-5 (PMC9098434; doi:10.1038/s41598-022-11716-5)
Supplement: Supplementary file 1 — Supplementary Information. [file 41598_2022_11716_MOESM1_ESM.docx]

| **Location** | **Latitude** | **Longitude** | **Total AGB (mg)** | **Total BGB (mg)** | **Total C (mg)** |
| --- | --- | --- | --- | --- | --- |
| Lothian Island | 21.648960 | 88.344489 | 68.57 | 15.57 | 28.67 |
| Burirdabri forest camp | 22.077813 | 89.029095 | 87.63 | 20.6 | 48.96 |
| Samsher nagar camp | 22.191429 | 89.060481 | 63.41 | 16.68 | 31.34 |
| Jhingakhali bit office | 22.019552 | 88.682970 | 92.57 | 21.89 | 51.57 |
| Sajnekhali wildlife park | 22.123759 | 88.832044 | 81.36 | 18.37 | 45.54 |
| Sudhanyakhali tiger camp | 22.101077 | 88.800824 | 83.57 | 19.74 | 48.17 |
| Dobanki camp | 21.988026 | 88.756692 | 91.48 | 20.67 | 50.27 |
| Netidhopani camp | 21.920388 | 88.746862 | 88.79 | 19.58 | 46.19 |
| Jambu dweep | 21.582007 | 88.185141 | 70.69 | 15.39 | 37.59 |
| Henry island | 21.586142 | 88.301484 | 93.59 | 22.07 | 52.74 |
| Bonnie camp | 21.830401 | 88.622345 | 95.36 | 23.23 | 54.39 |
| Jharkhali park | 22.016451 | 88.682958 | 65.28 | 12.26 | 34.29 |
| Average | - | - | 81.85 | 18.83 | 44.14 |

**Table S1. AGB (above ground biomass), BGB (below ground biomass) and total carbon storage determination in different location within the SBR region**

| **Space craft ID** | **Sensor ID** | **Date** | **Path** | **Row** | **Sun azimuth** | **Sun elevation** | **Band details** |
| --- | --- | --- | --- | --- | --- | --- | --- |
| Landsat | MSS | 12-05-1975 | 148 | 044 | 143.84 | 35.23 | Band 1 = Green (0.5-0.6 μm)  Band 2= Red (0.6-0.7 μm)  Band 3 = NIR (0.7-0.8 μm)  Band 4 NIR (0.8-1.1 μm) |
| Landsat | MSS | 12-05-1975 | 148 | 045 | 142.98 | 36.23 |  |
| Landsat | TM | 11-04-2000 | 138 | 044 | 143.21 | 46.86 | Band 1 = Blue (0.45–0.52 μm)  Band 2 = Green (0.52–0.60 μm)  Band 3 = Red (0.63–0.69 μm)  Band 4 = NIR (0.76–0.90 μm)  Band 5 = SWIR 1 (1.55–1.75 μm) Band 6 = Thermal (10.40–12.50 μm) Band 7 = SWIR 2 (2.08–2.35 μm |
| Landsat | TM | 11-04-2000 | 138 | 045 | 136.94 | 51.27 |  |
| Landsat  Landsat | OLI_TIRS  OLI_TIRS | 04-06-2020  04-06-2020 | 138  138 | 044  045 | 113.84  139.49 | 64.90  56.75 | Band 1= Coastal Aerosol (0.43–0.45 μm)  Band 2= Blue (0.45 –0.51 𝜇m)  Band 3 = Green (0.53–0.59 𝜇m)  Band 4 = Red (0.64–0.67 𝜇m)  Band 5 = NIR (0.85–0.88 𝜇m)  Band 6 = SWIR 1 (1.57–1.65 𝜇m)  Band 7 = SWIR 2 (2.11–2.29 𝜇m) Band 8 = Panchromatic (0.50–0.68 𝜇m)  Band 9 = Cirrus (1.36–1.38 𝜇m)  Band 10 = Thermal Infrared 1 (10.6–11.19 𝜇m)  Band 11 = Thermal Infrared 2 (11.5–12.51 𝜇m) |

**Table S2. Detailed descriptions of the used satellite images**

| **Ecosystem services** | **Ecosystem service values (USD /ha/year)** | | | | | |
| --- | --- | --- | --- | --- | --- | --- |
|  | **Waterbody** | **Settlement** | **Croplands** | **Mangrove forest** | **Sparse forest/open forest** | **Fallow land** |
| **Provisional service** |  |  |  |  |  |  |
| Water supply | 2117 | _ | _ | _ | _ | _ |
| Food production | 41 | _ | 54 | 466 | 67 | _ |
| Raw material | _ | _ | _ | 162 | _ | _ |
| Genetic resources | _ | _ | _ | _ | _ | _ |
| **Regulating services** |  |  |  |  |  |  |
| Water regulation | 5445 | _ | _ | _ | 3 | _ |
| Waste treatment | 665 | _ | _ | 6696 | 87 | _ |
| Erosion control | _ | _ | _ | _ | 29 | _ |
| Climate regulation | _ | _ | _ | _ | _ | _ |
| Biological control | _ | _ | 24 | _ | 23 | _ |
| Gas regulation | _ | _ | _ | _ | 7 | _ |
| Disturbance regulation | _ | _ | _ | 1839 | _ | _ |
| **Supporting services** |  |  |  |  |  |  |
| Nutrient Cycling | _ | _ | _ | _ |  | _ |
| Pollination | _ | _ | 14 | _ | 25 | _ |
| Soil formation | _ | _ | _ | _ | 1 | _ |
| Habitat/refuge | _ | _ | _ | 169 | _ | _ |
| **Cultural services** |  |  |  |  |  |  |
| Recreation | 230 | _ | _ | 658 | 2 | _ |
| Cultural services | _ | _ | _ | _ | _ | _ |
| Total | 8498 | 0 | 92 | 9990 | 244 | 0 |

**Table S3. Details of value coefficient for ecosystem services of different land use land cover classes (according to Costanza et al.^1^)**

| **LULC classes** | **Costanza et al.^1^** | **Costanza et al.^4^** | | **De Groot et al.^25^** | **Xie et al.^29^** |
| --- | --- | --- | --- | --- | --- |
|  | **C97a** | **C97b** | **C11** | **D12** | **X8** |
| Waterbody including rivers and creeks | 8498 | 11727 | 12512 | 4267 | 8377.85 |
| Settlement/build-up area | 0 | 0 | 6661 | 0 | 0 |
| Cropland | 92 | 126 | 5567 | 5567 | 1459.43 |
| Mangrove forest | 9990 | 13786 | 193843 | 193845 | 7079.17 |
| Sparse forest/ open forest | 232 | 321 | 4166 | 2871 | 2155.88 |
| Fallow land | 0 | 0 | 0 | 0 | 0 |

**Table S4. Ecosystem service values (USD ha^-1^ year^-1^) per unit area (ha) in Sundarban region by different unites of valuation.**

| **LULC** | **Million USD year^-1^** | | | | | | | | |
| --- | --- | --- | --- | --- | --- | --- | --- | --- | --- |
|  | **1975** | **%** | **2000** | **%** | **2020** | **%** | **1975-2000** | **2000-2020** | **1975-2020** |
| Waterbody including rivers and creeks | 2702.72 | 9.83 | 2935.36 | 10.96 | 3072.84 | 12.63 | 232.64 | 137.48 | 370.12 |
| Settlement/build-up area | 54.36 | 0.20 | 157.66 | 0.59 | 244.56 | 1.00 | 103.30 | 86.90 | 190.20 |
| Cropland | 845.34 | 3.07 | 780.53 | 2.91 | 745.77 | 3.06 | -64.81 | -34.76 | -99.57 |
| Mangrove forest | 23823.26 | 86.65 | 22857.56 | 85.31 | 20178.22 | 82.92 | -965.70 | -2679.35 | -3645.05 |
| Sparse forest/ open forest | 68.21 | 0.25 | 61.00 | 0.23 | 93.88 | 0.39 | -7.21 | 32.88 | 25.67 |
| Fallow land | 0.00 | 0.00 | 0.00 | 0.00 | 0.00 | 0.00 | 0.00 | 0.00 | 0.00 |

**Table S5. Changes of ESVs of various LULC classes**

| 1975 | **LULC type** | **Waterbody** | **Settlement** | **Cropland** | **Mangrove forest** | **Sparse forest** | **Fallow land** | **Total** | **Overall accuracy** |
| --- | --- | --- | --- | --- | --- | --- | --- | --- | --- |
|  | Waterbody | 43 | 3 | 3 | 0 | 1 | 1 | 51 | 84.4 |
|  | Settlement | 4 | 20 | 4 | 0 | 1 | 0 | 29 |  |
|  | Cropland | 0 | 3 | 52 | 1 | 2 | 1 | 59 |  |
|  | Mangrove forest | 0 | 1 | 0 | 60 | 0 | 0 | 61 |  |
|  | Sparse forest | 0 | 1 | 1 | 2 | 27 | 2 | 33 |  |
|  | Fallow land | 2 | 1 | 2 | 0 | 3 | 9 | 17 |  |
|  | Total | 49 | 29 | 62 | 63 | 34 | 13 | 250 |  |
| 2000 | **LULC type** | **Waterbody** | **Settlement** | **Cropland** | **Mangrove forest** | **Sparse forest** | **Fallow land** | **Total** | **overall accuracy** |
|  | Waterbody | 44 | 2 | 3 | 0 | 2 | 1 | 52 | 86 |
|  | Settlement | 3 | 28 | 3 | 1 | 1 | 1 | 37 |  |
|  | Cropland | 0 | 2 | 54 | 1 | 1 | 0 | 58 |  |
|  | Mangrove forest | 0 | 0 | 1 | 56 | 1 | 0 | 58 |  |
|  | Sparse forest | 0 | 2 | 0 | 1 | 28 | 1 | 32 |  |
|  | Fallow land | 3 | 1 | 2 | 0 | 2 | 5 | 13 |  |
|  | Total | 50 | 35 | 63 | 59 | 35 | 8 | 250 |  |
| 2020 | **LULC type** | **Waterbody** | **Settlement** | **Cropland** | **Mangrove forest** | **Sparse forest** | **Fallow land** | **Total** | **overall accuracy** |
|  | Waterbody | 46 | 2 | 1 | 0 | 1 | 0 | 50 | 86.8 |
|  | Settlement | 3 | 28 | 2 | 2 | 1 | 1 | 37 |  |
|  | Cropland | 0 | 2 | 56 | 1 | 2 | 0 | 61 |  |
|  | Mangrove forest | 0 | 0 | 1 | 49 | 0 | 1 | 51 |  |
|  | Sparse forest | 1 | 1 | 1 | 0 | 29 | 0 | 32 |  |
|  | Fallow land | 2 | 2 | 3 | 0 | 3 | 9 | 19 |  |
|  | Total | 52 | 35 | 64 | 52 | 36 | 11 | 250 |  |

**Table S6. Accuracy estimation of the land use land cover maps (1975, 2000 and 2020)**
